# Exclusive breastfeeding of Swedish children and its possible influence on the development of obesity: a prospective cohort study

**DOI:** 10.1186/1471-2431-8-42

**Published:** 2008-10-09

**Authors:** Karina Huus, Jonas F Ludvigsson, Karin Enskär, Johnny Ludvigsson

**Affiliations:** 1 Division of Pediatrics and Diabetes Research Centre, Linköping University, Sweden; 2 Department of Nursing Science, School of Health Sciences, Jönköping, Sweden; 3 Department of Pediatrics, Clinical Research Centre, Örebro University Hospital, Sweden; 4 Clinical Epidemiology Unit, Department of Medicine, Karolinska University Hospital, Sweden

## Abstract

**Background:**

Overweight and obesity are increasing among children all over the world. Socio-economic factors may influence the development of overweight and obesity in childhood, and it has been proposed that breastfeeding may protect against obesity. The aim of our study was to examine the relationship between exclusive breastfeeding and obesity when potential confounders, such as socioeconomic factors, are considered.

**Methods:**

The data analyzed was from ABIS (All Babies in Southeast Sweden), a prospective cohort study. All parents with children born between October 1, 1997 and October 1, 1999 in Southeast Sweden (n = 21,700) were asked to participate. Parents were asked to answer periodic questionnaires from the time of the child's birth (n = 16,058) until he/she was five years of age (n = 7,356). Cutoffs for overweight and obesity were defined according to Cole et al, age and gender adjusted. Short-term exclusive breastfeeding was defined as < 4 months of exclusive breastfeeding. Multiple logistic regressions were used to identify variables that predict the child's BMI (Body Mass Index) at five years of age.

**Results:**

At five years of age, 12.9% of the children in the study wereoverweight and 4.3% were obese. At the age of three months, 78.4% of the children were being breastfed exclusively. The median exclusive breastfeeding duration was four months. High maternal BMI > 30 (AOR = 1.07; CI = 1.05–1.09; P < 0.001), maternal smoking (AOR = 1.43; CI = 1.05–1.95; P = 0.023) and being a single parent (AOR = 2.10; CI = 1.43–3.09; P < 0.001) were associated with short-term exclusive breastfeeding (less than 4 months). Short-term exclusive breastfeeding was less common if one of the parents had a university degree (Mother: AOR = 0.74; CI = 0.61–0.90; P = 0.003 Father: AOR = 0.73; CI = 0.58–0.92; P = 0.008) or if the father was more than 37 years old (AOR = 0.74; CI = 0.55–0.99; P = 0.045). Short-term exclusive breastfeeding was associated with obesity in five-year-old children (simple logistic regression: OR = 1.44; CI = 1.00–2.07; P = 0.050), but when including other independent factors in the analysis, short-term exclusive breastfeeding did not attain statistical significance.

**Conclusion:**

We cannot exclude the possibility that exclusive breastfeeding influences weight development, but it does not seem to protect against obesity at five years of age.

## Background

Overweight and obesity are increasing among children all over the world [1-3]. The World Health Organization describes obesity as a global epidemic disease [4]. Many studies show that socioeconomic factors such as employment, family structure and parental education influence the development of overweight and obesity in childhood [5,6]. However, breastfeeding may also play a role in this development [7-10]. In Sweden, three out of four mothers exclusively breastfeed their child for at least two months [11], and put into an international perspective, the prevalence of breastfeeding is high [12]. Still, the prevalence of obesity is also increasing in Sweden [13,14]. Furthermore, data on what role breastfeeding plays in the development of obesity is inconsistent. Some studies show a protective effect [15-18], and others do not [19,20]. Most of these studies [15,18-20] are based on relatively small samples. In a systematic review of 69,000 children, breastfeeding seemed to protect against obesity [21]. However, an inverse association between breastfeeding duration (mostly never vs. never) and obesity was only observed in four of the nine studies analyzed in the review [21]. Therefore, further prospective studies with large samples are needed.

In an earlier study we found that certain socioeconomic factors such as level of parental education, paternal age, smoking habits, and maternal employment for less than three months during pregnancy, were positively associated with short-term exclusive breastfeeding (less than 4 months), while coffee consumption was not [22]. In another study, we also found that parental weight and level of education have an impact on the child's BMI (Body Mass Index) at five years of age [14].

The aim of the current study was to examine the relationship between exclusive breastfeeding and obesity when potential confounders, such as socioeconomic factors, are considered.

## Methods

### Participants and Design

All parents with children born between October 1, 1997 and October 1, 1999 in Southeast Sweden (n = 21,700) were asked to participate in the ABIS study (All Babies in Southeast Sweden), a prospective cohort study. The main purpose of the ABIS study was to examine the etiology of autoimmune diseases, especially Type 1 diabetes. Parents were asked to complete questionnaires at the time of the child's birth, and when the child was one, two and a half, and five years of age. The questionnaire response rate at infant birth was 74%, yielding a sample of 16,058 subjects (51.8% boys and 48.2% girls). The one year questionnaire was completed by 10,836 families, the two and a half year questionnaire by 8,763 families and the five year questionnaire by 7,356. Table 1 shows the baseline data for those 14,244 children from whom we had responses to all the questions, and the same baseline data for those 5,999 children whose parents, at the five-year follow-up, had responded to all the questions. This baseline data is then presented in the last column of Table 1 compared to the situation at the actual five-year follow-up, demonstrating that the material remains quite representative. The baseline values for parental age, level of education, country of birth, maternal smoking habits and parental civil status differ very little between the initial cohort of participants and those from the five-year follow-up. Mothers who dropped out of the study tended to be slightly younger and have a lower level of education, were often born in a foreign country, were often smokers and were often single parents at the time of birth.

**Table 1:** Baseline Characteristics for the total cohort at birth (n ≥ 14 244) and baseline data at birth for the cohort still participating at the five-year follow up (n ≥ 5 999) compared to the data for that group (≥ 5 999) at this follow-up.

	Baseline (n ≥ 14 244)	Baseline (n ≥ 5 999)	5 years (n ≥ 5 999)
Boys/girls, %	48.2/51.8	48.1/51.9	48.1/51.9
Overweight/obese, boys %	-	-	11.3/3.6
Overweight/obese, girls %	-	-	14.6/4.8
BMI mother, mean (sd)	23.8 (3.9)*	23.7 (3.9)*	24.1 (4.0)
BMI father, mean (sd)	25.0 (3.0)*	25.0 (2.9)*	25.6 (3.1)
Maternal age, mean (sd)	29.6 (4.6)	29.9 (4.5)	
Paternal age, mean (sd)	32.1 (5.5)	32.3 (5.4)	
University degree, mother %	31.7	34.5	39.4
University degree, father %	24.6	25.3	26.6
Smoking, mother %	11.1	7.8	10.7
Smoking, father %	-	-	9.3
Single parent %	2.1	1.2	6.6
Fathers born abroad %	7.2	5.2	
Mothers born abroad %	6.6	5.3	

Exclusive breastfeeding, median†	4.0	4.0	

5 999 is the minimum number of participants who answered the questions.

†Median duration in months

*Information collected at 1-year examination.

BMI = Body Mass Index

sd = Standard deviation

The most common reasons for not participating in the ABIS study were reported as "lack of time", "too many questions in the questionnaire" or "children do not want go give biological samples" (which is part of ABIS) [14,23,24].

The 150-item questionnaires were distributed by the nurses at the Child Health Service. For each question, all existing data were used, even if some participants did not respond to every question. This explains why the number of participants differs between the different analyses.

### Data collection and analysis

Questions were asked concerning breastfeeding, introduction of infant formula or gruel, introduction of cow's milk, child and parental weight, and regarding various socioeconomic variables such as civil status, parental age, education and smoking habits, and whether or not the parents were born outside Sweden. The questions about exclusive breastfeeding, introduction of infant formula or gruel and the introduction of cow's milk had response alternatives: <1 month to > 8 months. For the question regarding breastfeeding, the parents stated how many months after birth the mother stopped breastfeeding the child.

Cutoffs for overweight and obesity at five years of age were defined according to Cole et al [25], age and gender adjusted. All weight and height data used in the study were received from the parents. However, these weights and heights were validated against those recorded at the Child Health Service. This validation showed a high correlation between weight and height, having an intraclass correlation coefficient of P < 0.001.

The following covariates were used for the logistic regression: family situation (single-cohabitant), maternal age (≤ 34 years at child birth vs. ≥ 35 years), paternal age (≤ 36 years vs. ≥ 37 years), number of siblings (0 vs. ≥ 1), maternal BMI (≤ 24.99 vs. ≥ 25), paternal BMI (≤ 24.99 vs. ≥ 25) and maternal and paternal education (university degree: no vs. yes).

When maternal age was used in the analysis, 35 years, commonly used in other medical contexts, was used as the cutoff age [26,27]. Fathers were, on average, two years older than mothers, and therefore 37 years was used as the paternal cutoff age. Short-term exclusive breastfeeding was defined as < 4 months of exclusive breastfeeding.

### Statistics

SPSS [28] was used for the statistical analyses. Correlations were assessed by using Pearsons correlation. Intra class correlation coefficient were used in validation of weights and heights. Simple and multiple logistic regressions were used to identify variables that predicted the child's BMI at five years of age. P-values < 0.05 were considered statistically significant.

The study was approved by the Research Ethics Committee at the Faculty of Health Sciences, Linköping University, Sweden.

## Results

### Description of breastfeeding prevalence

Questionnaire data one year after birth showed that at the age of three months 78.4% of the children were exclusively breastfed. The median exclusive breastfeeding duration, which was similar for boys and girls, was four months (1 = 4, 3 = 6). The median age at the introduction of infant formula or gruel was five months (1 = 3, 3 = 7). The median age of children when they received cow's milk for the first time was six months (1 = 5, 3 = 7).

The median duration of any breastfeeding, according to questionnaire answers two and a half years after birth, was eight months (1 = 6, 3 = 11).

### Risk factors for any breastfeeding duration

Both maternal (R^2 ^= 0.028, P < 0.001) and paternal age (R^2 ^= 0.028, P < 0.001) were positively associated with the duration of any breastfeeding. Older mothers breastfed for a longer period of time than did younger mothers.

### Parental BMI and exclusive breastfeeding duration

Parental BMI >30 was associated with short-term exclusive breastfeeding (Father: Year five questionnaire Odds Ratio (OR) = 1.71; 95% CI = 1.31–2.21; P < 0.001; Mother: Year five questionnaire OR = 2.20; 95% CI = 1.72–2.82; P < 0.001).

### Risk factors for short-term exclusive breastfeeding

The main logistic regression showed that short-term exclusive breastfeeding was positively associated with maternal BMI >30 (Adjusted Odds Ratio; AOR = 1.07; 95% CI = 1.05–1.09; P < 0.001) and with maternal smoking (AOR = 1.43; 95% CI = 1.05–1.95; P = 0.023). Short-term exclusive breastfeeding was more often reported by single parents (AOR = 2.10; 95% CI = 1.43–3.09; P < 0.001).

Short-term exclusive breastfeeding was less common if one of the parents had a university degree (Mother: AOR = 0.74; 95% CI = 0.61–0.90; P = 0.003, Father: AOR = 0.73; 95% CI = 0.58–0.92; P = 0.008) or if the father was over 37 years old (AOR = 0.74; 95% CI = 0.55–0.99; P = 0.045) (table 2).

**Table 2:** Factors related to short-term exclusive breastfeeding (less than 4 months)^b^.

Independent variable			
	AOR	95% CI	P
Fathers BMI>30^c^	1.00	0.99–1.01	0.330
Mothers BMI>30^c^	1.07	1.05–1.09	<0.001
Single parent^c^	2.10	1.43–3.09	<0.001
Father >37 yrs old ^a^	0.74	0.55–0.99	0.045
Mother >35 yrs old ^a^	1.10	0.80–1.51	0.542
Mother smoking ^c^	1.43	1.05–1.95	0.023
Father smoking^c^	0.88	0.63–1.23	0.452
Mother with university degree ^c^	0.74	0.61–0.90	0.003
Father with university degree ^c^	0.73	0.58–0.92	0.008
Father born abroad ^a^	1.14	0.75–1.71	0.546
Mother born abroad ^a^	0.85	0.54–1.35	0.492

N = 3386

BMI = Body Mass Index

AOR = Adjusted Odds Ratio

^a^. The question was answered when the child was born.

^b^. The question was answered when the child was 1 year old.

^c^. The question was answered when the child was 5 years old.

### Obesity at five years of age

According to Cole et al, 12.9% of the children were overweight and 4.3% were obese at five years of age [25].

There was a weak association between short-term exclusive breastfeeding and obesity in five-year-old children (simple logistic regression: OR = 1.44; 95% CI = 1.00–2.07; P = 0.050). In the multivariate analysis, when other independent factors were included, this association did not attain statistical significance. In the multivariate analysis, obesity at five years of age was only associated with maternal smoking (AOR = 1.72; 95% CI = 1.00–2.93; P = 0.048) (Table 3).

**Table 3:** Factors related to risk of obesity at 5 years of age.

**Independent variable**			
	AOR	95% CI	P
Exclusive breastfeeding ^b ^(Less than 4 month)	1.22	0.81–1.83	0.341
Mothers age >35^a^	1.14	0.63–2.05	0.672
Fathers age >37^a^	1.09	0.65–1.85	0.735
Single parent ^c^	0.59	0.23–1.48	0.259
Mother born abroad ^a^	1.45	0.69–3.07	0.325
Father born abroad ^a^	1.58	0.79–3.16	0.192
Mother smoking ^c^	1.72	1.00–2.93	0.048
Father smoking ^c^	0.97	0.52–1.81	0.921
Mother with University degree ^c^	0.78	0.53–1.15	0.210
Father with University degree ^c^	0.77	0.49–1.22	0.269

N = 3654

BMI = Body Mass Index

AOR = Adjusted Odds Ratio

^a^. The question was answered when the child was born.

^b^. The question was answered when the child was 1 year old.

^c^. The question was answered when the child was 5 years old.

## Discussion

Obesity is quite common in Swedish children, and we found that 12.9% of the five year old children were overweight and 4.3% were obese. It has been proposed that long-term breastfeeding may protect against obesity [15-19,29,30]. Both metabolic imprinting and behavioral aspects may potentially explain this association [31]. In Sweden, most mothers breastfeed their children [11]. In our study, 78.4% of the mothers exclusively breastfed for at least three months. The median duration of any breastfeeding was eight months. In comparison with other countries these figures are quite high [12]. The common and long-term breast-feeding duration could therefore be expected to reduce the risk of obesity in Swedish children. Thus, the aim of our study was to analyze if exclusive breastfeeding protects against obesity, when adjusting for a number of confounding factors.

Although we found a trend indication that short-term exclusive breastfeeding was in fact associated with obesity at five years of age (P = 0.05), this association did not remain statistically significant when other factors, such as parental smoking, education, parental country of birth, civil status and parental age, were included in the analysis. Lack of association can always be caused by methodological problems. Our results are based on a very large number of children followed prospectively from birth, which should allow us to include several confounding factors in our analysis, and we do not believe that low power was the reason for a lack of observed associations between breastfeeding and obesity, if breastfeeding has a clinically important protective effect.

The weight and height of the children were reported by the parents, and earlier validation has shown this to be a reliable source.

In this study, BMI cutoffs, age and gender adjusted according to Cole at al [25], were used as the outcome variable for obesity and whether or not this is the best parameter can be discussed. In another study dual energy x-ray absorptionmetry was used and a protective effect of breastfeeding against obesity was found [17].

One weakness of the current study is the dropout rate. We cannot exclude the risk of selection bias since mothers responding to the five-year questionnaire were slightly older and were often more highly educated than mothers completing the baseline questionnaire. This may, to some extent, explain why mothers in this study report a longer breastfeeding duration, both exclusive and any, than mothers in previous Swedish studies [11].

In a multivariate analysis, only maternal smoking (AOR = 1.72; CI = 1.00–2.93; P = 0.048) remained significantly associated with obesity at five years of age, which indicates that there may be other social factors to be considered when trying to prevent obesity among children.

## Conclusion

Our conclusion is that although there still is a possibility that breastfeeding influences weight development, exclusive breastfeeding does not seem to protect against obesity, at least not in early childhood. Breastfeeding should be encouraged for several different reasons, but for prevention of obesity in Swedish children other measures may be more important.

## Competing interests

The authors declare that they have no competing interests.

## Authors' contributions

KH was responsible of the final analyses, outcome assessments and the writing of the manuscript. JFL supervised the statistics and the manuscript writing. KE supervised the manuscript writing. JL had the primary responsibility for the study design, and supervised the execution of the study, the data analysis and the manuscript writing.

## Pre-publication history

The pre-publication history for this paper can be accessed here:


